# The sweating process promotes toxigenic fungi expansion and increases the risk of combined contamination of mycotoxins in *Radix Dipsaci*

**DOI:** 10.3389/fmicb.2024.1394774

**Published:** 2024-06-06

**Authors:** Yangyan Ge, Lulu Wang, Dapeng Su, Qingsong Yuan, Chenghong Xiao, Min Hu, Chuanzhi Kang, Lanping Guo, Tao Zhou, Jinqiang Zhang

**Affiliations:** ^1^Guizhou University of Traditional Chinese Medicine, Guiyang, China; ^2^Resource Institute for Chinese and Ethnic Materia Medica, Guizhou University of Traditional Chinese Medicine, Guiyang, China; ^3^State Key Laboratory of Dao-di Herbs, Beijing, China

**Keywords:** *Radix Dipsaci*, sweating, mycobiota, toxigenic fungi, mycotoxins

## Abstract

Sweating is one of the most important processing methods of Chinese medicinal herbs. However, the high temperature and humidity environment required for sweating Chinese medicinal herbs makes it very easy for fungi to breed, especially toxigenic fungi. The mycotoxins produced by these fungi will then contaminate the Chinese medicinal herbs. In this study, we explored the changes in mycobiota, toxigenic fungi, and mycotoxins with and without sweating in *Radix Dipsaci* (RD), a typical representative of traditional Chinese medicine that requires processing through sweating. We also isolated and identified the toxigenic fungi from RD, whether they were subjected to sweating treatment or not, and examined their toxigenic genes and ability. The results showed that the detection rate of mycotoxins (aflatoxins, ochratoxins, zearalenone, and T-2 toxin) in RD with sweating was 36%, which was 2.25-fold higher than that in RD without sweating. We also detected T-2 toxin in the RD with sweating, whereas it was not found in the RD without sweating. The sweating process altered the fungal composition and increased the abundance of *Fusarium* and *Aspergillus* in RD. *Aspergillus* and *Fusarium* were the most frequently contaminating fungi in the RD. Morphological and molecular identification confirmed the presence of key toxigenic fungal strains in RD samples, including *A. flavus, A. westerdijkiae, F. oxysporum* and *F. graminearum*. These four fungi, respectively, carried *AflR*, *PKS*, *Tri7*, and *PKS14*, which were key genes for the biosynthesis of aflatoxins, ochratoxins, zearalenone, and T-2 toxin. The toxigenic ability of these four fungal strains was verified in different matrices. We also found that *A. flavus*, *A. westerdijkiae*, and *F. oxysporum* were isolated in RD both with sweating and without sweating, but their isolation frequency was significantly higher in the RD with sweating than in the RD without sweating. *F. graminearum* was not isolated from RD without sweating, but it was isolated from RD with sweating. These findings suggest that the sweating process promotes the expansion of toxigenic fungi and increases the risk of combined mycotoxin contamination in RD.

## Highlights

- The sweating process altered the fungal composition and increased the abundance of *Fusarium* and *Aspergillus* in *Radix Dipsaci*.
- Sweating process increases the risk of combined contamination of mycotoxins in *Radix Dipsaci*.
- Mycotoxins in *Radix Dipsaci* are mainly derived from *Aspergillus flavus*, *Aspergillus westerdijkiae*, *Fusarium oxysporum*, and *Fusarium graminearum*.

## 1 Introduction

Mycotoxins are toxic secondary metabolites produced by toxigenic fungi under suitable conditions, which are commonly found in a variety of crops, including traditional Chinese herbs (Su et al., 2018). Molecular methods were used to test whether these genes were expressed in fungi, and then the potentially toxigenic fungi were preliminarily screened. The processes of cultivation, harvesting, storage and processing, traditional Chinese herbs may be contaminated by mycotoxins, which may affect the quality and safety of Chinese herbal medicines Currently, the main mycotoxins susceptible to fungal contamination in traditional Chinese herbs include aflatoxins (AFs), ochratoxins (OTs), T-2 toxin, zearalenone (ZEN), and deoxynivalenol (DON) (Diana Di Mavungu et al., 2009; Han et al., 2012). These mycotoxins may have various chronic and acute effects, including hepatotoxicity, genotoxicity, immunosuppression, estrogenic effects, nephrotoxicity, teratogenic effects, and carcinogenic effects. Therefore, strengthening the monitoring and control of mycotoxin pollution in Chinese medicinal materials is of great significance for ensuring the quality and safety of Chinese medicinal materials and maintaining people’s health. Different toxigenic fungi possess specific key toxigenic genes (Moretti et al., 2013; Ben et al., 2024). Molecular methods were used to test whether these genes were expressed in fungi, and then the potentially toxigenic fungi were preliminarily screened (Alsalabi et al., 2023). The potential toxigenic fungi were further confirmed through qualitative and quantitative analysis of specific mycotoxins in their metabolites (Ben et al., 2024).

Sweating is a special processing method used in the drying and processing of certain medicinal materials, such as roots, rhizomes, and cortex (Jin-Ao et al., 2013). It promotes the release of internal moisture from these medicinal parts and facilitates their drying (Jin-Ao et al., 2013; Wang et al., 2016). The high temperature and humidity environment and the relatively long processing time required during the sweating process contribute to the breeding and growth of fungi, especially some toxigenic fungi (Deng et al., 2020; Liao et al., 2020; Wicaksono and Illes, 2022). The mycotoxins produced by them will contaminate the Chinese medicinal herbs. At present, the effects of sweating on the secondary metabolites of Chinese medicinal herbs have been widely reported (Wu et al., 2019; Cao et al., 2020; Chen et al., 2021; He et al., 2023). However, there are few reports on the effect of sweating on contamination by toxigenic fungi and mycotoxins in Chinese medicinal herbs.

The *Radix Dipsaci* (RD) is the dried root of the plant *Dipsacus asper* Wall. ex Henry, which represents traditional Chinese medicine and requires a sweating process. RD is commonly used in the treatment of hepatic or renal disorders, osteoporosis, tendon damage, and uterine hemorrhage due to its potent therapeutic properties (Ke et al., 2016; Zhang et al., 2019; Skala and Szopa, 2023). Here, we detected mycotoxins using ultra-high performance liquid chromatography followed by tandem mass UPLC–MS/MS and investigated the changes in mycobiota, toxigenic fungi, and mycotoxins in RD before and after sweating. We also isolated and identified the toxigenic fungi from RD, whether they were subjected to sweating treatment or not, and examined their toxigenic genes and ability. This study will clarify the effect of the sweating process on toxigenic fungi in Chinese medicinal herbs and provide a basis for preventing the pollution of toxic fungi during sweating processing of Chinese medicinal herbs.

## 2 Materials and methods

### 2.1 Preparation of samples

*Dipsacus asper* were planted in plant nursery at Guizhou University of Traditional Chinese Medicine for 3 years and their roots were collected in October 2022. Fresh samples were randomly divided into two parts (25 samples for each group), one of which did not undergo sweating processing as a control group, and the other part processed according to the sweating method established in our previous study (Zhou Tai-min, 2021). Briefly, the samples required for sweating were dried in a dryer until the relative water content reached 40% and sweated for 3 days at 25°C in an airtight environment. These two sets of samples were stored in a −80°C freezer for detection of mycotoxins, mycobiota sequencing and isolation and identification of toxigenic fungi simultaneously.

### 2.2 Mycobiota sequencing and analysis

DNA extraction of RD and its amplification refer to the previous report (He et al., 2023). The ITS regions of the fungal 18S rRNA genes were amplified with primers ITS1F (5′-CTTGGTCATTTAGAGGAAGTAA-3′) and ITS2R (5′-GCTGCGTTCTTCATCGATGC-3′). PCR was performed using a 20 μL reaction system consisting of 2 μL 10 × Buffer, 2 μL 2.5 mM dNTPs, 0.8 μL ITS1F/IT2R (5 μM), 0.2 μL rTaq polymerase, 0.2 μL BSA, 10 ng DNA template, 2 μL RTAQ polymerase, and ddH_2_O to a final volume of 20 μL. The whole process was carried out in the PCR amplifier (ABI Gene Amp 9,700, United States), and the procedure was as follows: pre-denaturation at 95°C for 3 min; Denatured at 95°C for 30 s, annealed at 55°C for 30 s, extended at 72°C for 45 s, and expanded for 35 cycles. It was extended for 10 min at 72°C. The PCR products were detected through 2% agarose gel electrophoresis. Purified amplicons were pooled in equimolar ratios and paired-end sequenced on an Illumina MiSeq platform (Illumina, San Diego, United States) according to the standard protocols described by Majorbio Bio-Pharm Technology Co. Ltd. (Shanghai, China). The raw sequencing reads were deposited into the NCBI Sequence Read Archive (BioProject ID PRJNA1085876).

Sequences in the library were clustered using USearch, operational taxonomic units (OTUs) were clustered at 97% similarity for non-repetitive sequences (excluding individual sequences), and chimeras were removed during clustering to obtain representative sequences of OTUs (Magoc and Salzberg, 2011; Schmieder and Edwards, 2011; Zhang et al., 2014). Based on the OTUs information, rarefaction curves and alpha diversity indices including observed OTUs, Ace, Chao richness, and Shannon index were calculated with Mothur v1.30.1. The similarity among the microbial communities in different samples was determined by principal coordinate analysis (PCoA) based on Bray–Curtis dissimilarity using the Vegan v2.5-3 package.

### 2.3 Detection of mycotoxins

#### 2.3.1 Sample pretreatment

All the samples were dried until their moisture content was reduced to less than 10%, and then they were also ground and passed through a 50-mesh sieve for subsequent detection. Herb powder (2.0 g) was mixed with 20.0 mL of acetonitrile: water: formic acid (80: 19:1) and shaken for 1 h at 180 rpm. MgSO_4_ (2 g) and NaCl (1 g) were added to the mixture, which was swirled for 1 min, then centrifuged at 5000 rpm for 5 min. Supernatant (5 mL) was added to a mixture of the following pure powders, which served as “cleaning agents” to remove lipids, pigments, organic acids and sugars (Zhai et al., 2023): Octadecyl trichlorosilane (C_18_) (0.3 g), primary secondary amine (PSA, 0.1 g), MgSO_4_ (0.3 g) and graphitized carbon black (GCB, 0.01 g). The mixture was swirled for 1 min, then an aliquot of supernatant (3 mL) was transferred to a new tube and evaporated nearly to dryness at 40°C under nitrogen. The residue was reconstituted with 1.0 mL 50% acetonitrile in water, filtered through a membrane with pores of diameter 0.22 μm, and used in the analytical procedure described below.

#### 2.3.2 Preparation of standard solution

The following mycotoxin standards of purity ≥99% were purchased from Pribolab (Qingdao, Shandong, China) and stored at −20°C in the dark: aflatoxin B1 (catalog no. MSS1003), aflatoxin B2 (MSS1014), aflatoxin G1 (MSS1005), aflatoxin G2 (MSS1006), ochratoxin A (MSS1020), zearalenone (MSS1024) and T-2 toxin (MSS1023). Stock solutions of all mycotoxins were prepared by dissolving the powders in 50% (v/v) acetonitrile in water to final concentrations of 1.25 mg/mL in the case of zearalenone or 1 mg/mL in the case of the others. Aliquots of 0.10–10.0 mL of these stock solutions were used to prepare working solutions of 12.5 μg/mL in the case of zearalenone or 10 μg/mL in the case of the others. Aliquots of 1.0–25 mL from these working solutions were mixed to prepare a standard cocktail containing zearalenone at 500 ng/mL and all other mycotoxins at 400 ng/mL.

#### 2.3.3 Detection of mycotoxins

We detected all seven mycotoxins using ultra-high performance liquid chromatography followed by tandem mass ultra-high performance liquid chromatography-mass spectrometry (UPLC-MS/MS) using a QTRAP 5500 quadrupole system (SCIEX, United States) as the methods of Fan et al. (2022). Chromatographic parameters were as follows: column type, Waters HSS T3 (100 × 2.1 mm, 1.8 μm); column temperature, 40°C; mobile phases, 0.1% formic acid in water (solvent A) and acetonitrile (solvent B); flow rate, 0.3 mL/min; and sample volume, 5 μL. The following elution gradient was performed: 0–3 min, 85% A; 3–5 min, 65% A; 5–7 min, 55% A; 7–7.5 min, 50% A. The mass spectrometer was optimized in the multiple reaction monitoring mode, and the positive electrospray ionization [ESI^+^] polarity was employed. The capillary voltage, ion source temperature, cone hole blowback flow rate, desolvation temperature, and solvent gas flow rate were 3.5 kV, 150°C, 30 L·h−1, 500°C, and 600 L·h−1, respectively (Han et al., 2010). Data was collected in the multi-reaction monitoring (MRM) mode, and the mass spectrum parameters of each mycotoxin reference solution were shown in Table 1.

**Table 1:** Mass spectrum parameters of the seven mycotoxins.

Reference name	Parent ion m/z	Daughter ionm/z	Cone-hole voltage/eV	Collision voltage/eV	Acquisition mode
AFB_1_	313.1	285.0^1)^/241.0^2)^	30	25/30	[M + H]^+^
AFB_2_	315.1	287.0^1)^/259.0^2)^	30	25/20	[M + H]^+^
AFG_1_	329.1	243.1^1)^/283.1^2)^	30	25/20	[M + H]^+^
AFG_2_	331.1	245.0^1)^/257.0^2)^	30	30/28	[M + H]^+^
OTA	404.1	238.9^1)^/301.0^2)^	20	15/25	[M + H]^+^
T-2	489.0	245.0^1)^/327.0^2)^	22	28/22	[M + Na]^+^
ZEN	319.1	283.0^1)^/301.0^2)^	10	15/10	[M + H]+

#### 2.3.4 Validation of the analytical method for mycotoxin detection

We validated the analytical approach in Section 2.3.3. First, we prepared the test sample and the mycotoxin reference solution according to the above method for injection. The blank disyllabic sample extract was selected to dilute the mixed standard into a series of concentrations to establish a standard curve. The signal-to-noise ratio S/N ≥ 3 was defined as the limit of detection (LOD), and S/N ≥ 10 was defined as the limit of quantification (LOQ). Six parallel sample solutions were tested, and the repeatability was investigated based on the determined relative standard deviation of mycotoxin content. Stability was assessed by calculating the relative standard deviation of mycotoxin content measured at 0, 2, 4, 6, 12, and 24 h. The precision of the instrument was evaluated by calculating the relative standard deviation of the mycotoxin peak area detected in six consecutive injections. The recovery rate was investigated using the blank spiked recovery method. The test solution of blank RD was prepared according to the method described above. The solution was combined with “low” (the concentration of ZEN is 100 ng/mL, and the concentration of other mycotoxins is 80 ng/mL), “medium” (the concentration of ZEN is 200 ng/mL, and the concentration of other mycotoxins is 80 ng/mL), and “high” (ZEN concentration of 300 ng/mL, and the rest of the fungus toxin concentration at 240 ng/mL) in a mixture containing mycotoxin standard products at different concentrations. Each concentration was performed in triplicate, resulting in a total of nine samples. Mycotoxin recovery and its relative standard deviation were determined.

### 2.4 Isolation and identification of toxigenic fungi

The samples of RD without sweating and with sweating were first cut into tissue blocks measuring 5 mm × 5 mm × 5 mm. The toxigenic fungi were then separated using the tissue block method (Song et al., 2022). After the colonies grew out from the tissue blocks, the fungi were purified through single spore separation and translocated multiple times until a single colony was formed. They were stored at 4°C on a potato dextrose agar medium. The isolated fungi were identified morphologically according to the Atlas of Clinical Fungi (de Hoog, 2000). Colony morphology was observed on potato dextrose agar medium, including colony shape, size, color, mycelia type, spore production, and growth on the medium.

The mycelium of fungi cultured on potato dextrose agar medium was collected for molecular identification, and the DNA was extracted using a lysis buffer containing hexadecyltrimethylammonium bromide as described by Sadhasivam et al. (2017). Then the DNA extracted from each strain was used as a template to amplify the ITS2 gene using universal primers ITS1 (5’-TCCGTAGGTGAACCTGCGG-3′) and ITS4 (5’-TCCTCCGCTTATTGATATGC-3′) (Frimpong et al., 2019). The PCR reaction was performed using a 20 μL reaction system with the same amplification procedure as described in Section 2.3. Purified amplicons were pooled in equimolar ratios and subjected to paired-end sequencing (2 × 300) on an Illumina MiSeq platform (Illumina, San Diego, United States) following the standard protocols provided by Majorbio Bio-Pharm Technology Co. Ltd. Shanghai, China). The obtained sequences were analyzed using the BLAST program^1^ provided by the NCBI website to determine the genera and species of the fungal isolates (Wu et al., 2013). The construction of a neighbor-joining tree was performed based on multiple sequence alignment using MEGA 5.0 with bootstrap replications (Tamura et al., 2011).

### 2.5 Detection of toxigenic genes

In the analysis of key genes involved in toxin synthesis, we selected conserved regions of AFs, OTA, T-2, and ZEN to design specific primers for the synthesis of key genes *AflR*, *PKS*, *Tri7*, and *PKS14*. PCR was used to detect the presence of *AflR*, *PKS*, *Tri7*, and *PKS14* in the purified fungi by using the DNA of each fungus as a template. The primers are listed in Table 2. The PCR reaction was performed using a 20 μL reaction system with the same amplification procedure as described in section 2.4.

**Table 2:** Primers sequence.

Toxin type	Gene	Sequence (5′-3′)	Fragment size
AFs	*AflR*	GCACCCTGTCTTCCCTAACA	400 bp
		ACGACCATGCTCAGCAAGTA	
OTA	*PKS*	GCCAGACCATCGACACTGCATGCTC	536 bp
		CGACTGGCGTTCCAGTACCATGAGC	
T-2	*Tri7*	GCGAGGTATTGGAACRCCATG	667 bp
		TCTTCGATAATAATRCCGACAA	
ZEN	*PKS14*	CCCTCGCCAAGCACCTCATC	695 bp

### 2.6 Toxin production verification

The isolated toxigenic fungi were further inoculated on autoclaved rice and RD, which had been adjusted to 30% water content and cultured at 25°C for 14 days. After drying at 45°C, they were crushed. 2 g of rice and RD were weighed separately and mixed with 10 mL of a 70% methanol solution. The mixture was then centrifuged, and 5 mL of the supernatant was collected. The volume was adjusted to 10 mL, followed by elution of the immunoaffinity column with 1.0 mL of methanol. Finally, detection was performed using an overused 0.22 μm filter membrane according to the same method as described in section 2.3.

## 3 Results

### 3.1 Validation of the analytical method

**Figure 1:**
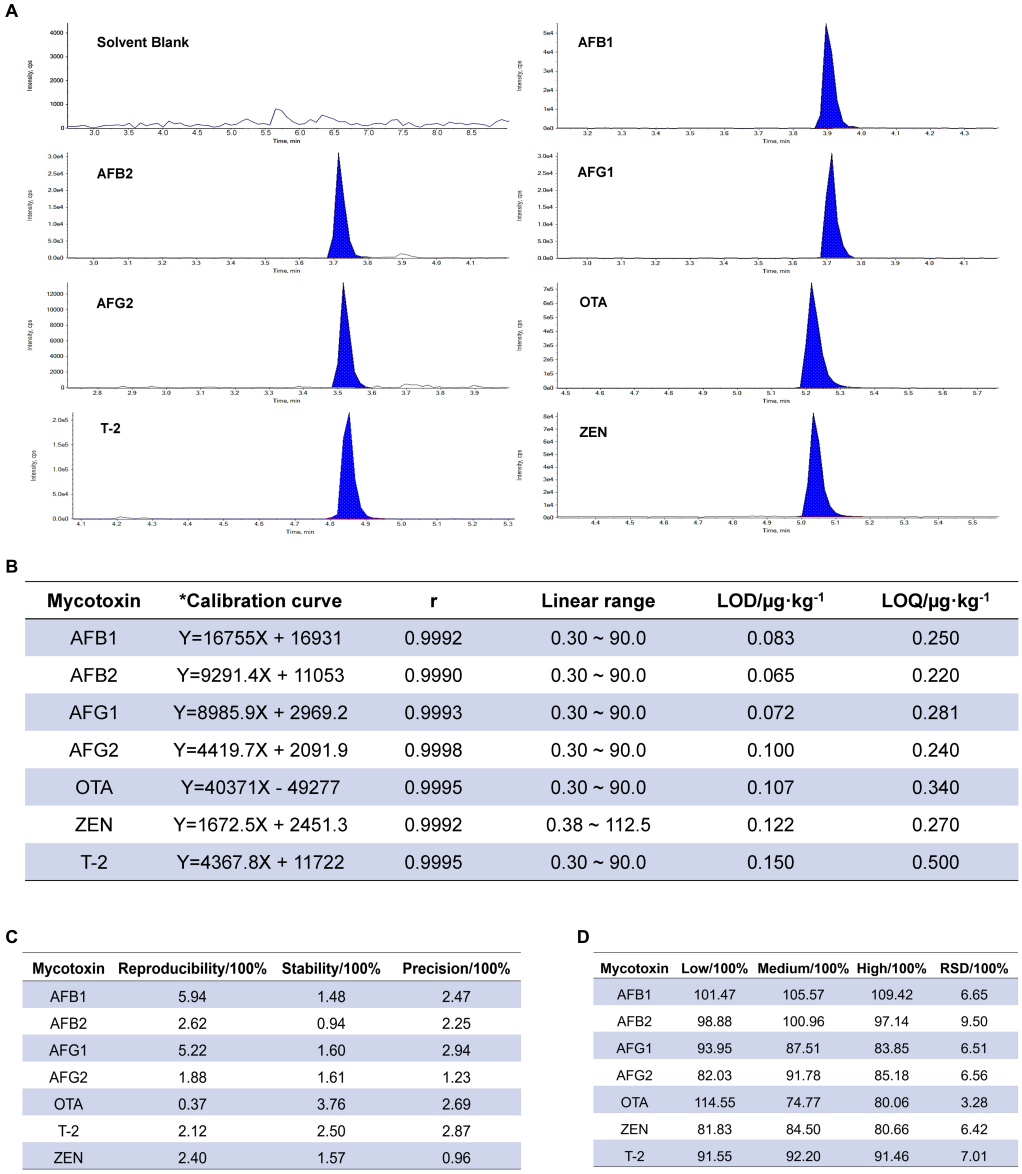
Validation of the analytical method. **(A)** Specific chromatogram of seven mycotoxins in *Radix Dipsaci*. cps, counts per second. **(B)** Calibration and validation of the analytical method for simultaneous detection of mycotoxins in *Radix Dipsaci*. * Calibration curves were adjusted for matrix effects (see Methods). LOD, limit of detection; LOQ, limit of quantitation. **(C)** Repeatability, stability and precision for determination of mycotoxins in *Radix Dipsaci*. **(D)** Recovery of seven kinds of mycotoxins from *Radix Dipsaci*. RSD, relative standard deviation.

In the investigation of specificity, the chromatographic peak of mycotoxin was well separated, and there was no interference at the corresponding position of the blank group, indicating that the method had good specificity (Figure 1A). The linear relationship results showed a good linear range (*r* ≥ 0.9990) for the seven mycotoxins. The detection limits of 7 mycotoxins were 0.065–0.230 μg kg^−1^, and the limits of quantification were 0.22–0.50 μg kg^−1^ (Figure 1B). The repeatability RSD was 0.37–5.94% (Figure 1C); the RSD value of 24 h stability was 0.94–3.76% and less than 3%. In the precision test, the RSD of precision was 0.74–2.94%, were lower than 15% of the requirements of Chinese Pharmacopoeia (2020 edition), indicating that the method had good repeatability, stability and precision. The results of precision testing indicated that the RSD ranged from 0.74 to 2.94% (Figure 1C), which was lower than the requirement of 15% stated in the Chinese Pharmacopoeia (2020 edition). Recoveries ranging from 73.30 to 119.40% of all tested mycotoxins with RSD lower than 15% (Figure 1D) were in line with the provisions of the Chinese Pharmacopoeia (2020 edition), indicating that the method was accurate and could be used for detecting mycotoxins in RD.

### 3.2 Sweating process altered the fungal composition and increased the abundance of *aspergillus* and *fusarium* in RD

**Figure 2:**
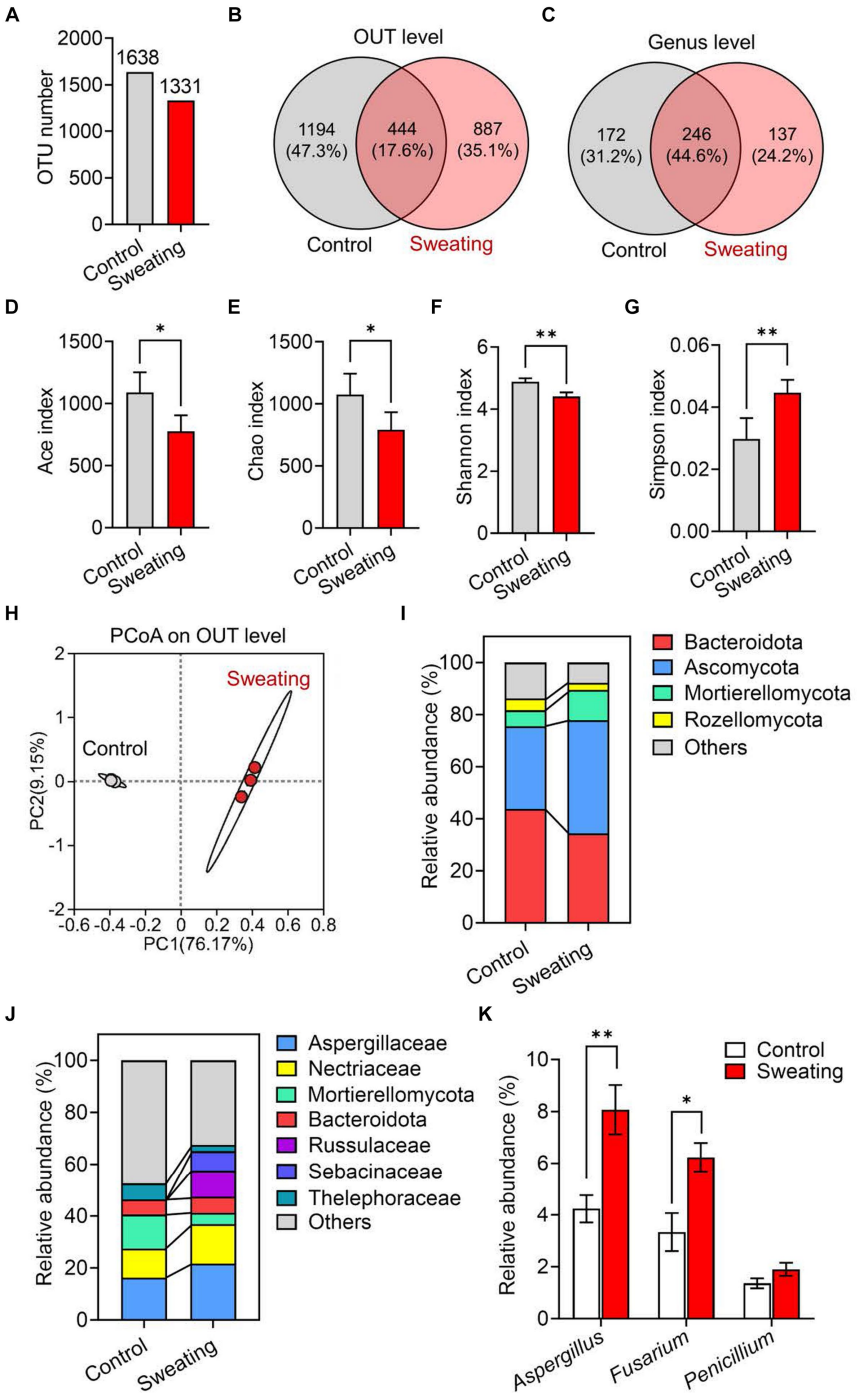
Mycobiota analysis of RD before and after sweating process. **(A)** Change in the number of operational taxonomic units (OTU) of fungi in RD before and after sweating process. **(B,C)** Wayne’s analysis of OTU and genus of fungi in RD before and after sweating process. **(D–G)** Alpha diversity at the level of OTUs in fungal communities in RD and after sweating process. Microbial diversity was quantified using Chao’s, Ace’s, Shannon’s and Simpson’s diversity indices (*n* = 3). **(H)** Principal component analysis (PCoA) of beta diversity in fungal communities in RD before and after sweating process, based on Bray–Curtis distances (*n* = 3). **(I,J)** Relative abundances of **(I)** phyla and **(J)** families of fungi in RD before and after sweating process. **(K)** Relative abundances of *Aspergillus* spp., *Fusarium* spp. and *Penicillium* spp. In RD before and after sweating process.

We prepared RD samples of sweating and non-sweating and conducted mycobiota sequencing. We observed a lower number of fungal OTUs in the RD with sweating compared to the RD without sweating (Figure 2A). Additionally, we identified 887 unique OTUs and 137 unique genera in the RD with sweating, which were not present in the RD without sweating (Figures 2B,C). The alpha diversity of fungi was analyzed by calculating the Chao, Ace, Shannon, and Simpson indices at the OTU level. The results showed that the Ace and Chao indices of fungi in RD with sweating were significantly decreased compared to those of the control group (Figures 2D,E), indicating that the sweating process reduced the richness of fungi in RD. The Shannon index of fungi significantly decreased, and the Simpson index significantly increased in RD with sweating when compared to the control group (Figures 2F,G), indicating that the sweating process reduced fungal diversity in RD.

Beta diversity was analyzed through principal coordinate analysis (PCoA) plots using nonphylogenetic Bray–Curtis metrics to assess differences in microbial composition (OTU) between the two groups (Figure 2H). There was a clear separation between the control and sweating groups along the first principal component (PC1) axis (Adonis *p* value = 0.001), suggesting that the sweating process can change the structure of the fungal community. For instance, at the phylum level, there was a significant increase in the abundance of *Ascomycota* and *Mortierellomycota* fungi after sweating, while the abundance of *Bacteroidota* and *Rozellomycota* fungi decreased significantly (Figure 2I). At the family level, we observed a significant increase in the relative abundance of *Aspergillaceae* and *Nectriaceae* fungi in RD with sweating compared to RD without sweating. The relative abundance of *Mortierellaceae* and *Thelephoraceae* fungi was significantly decreased in RD with sweating compared to RD without sweating (Figure 2J). *Fusarium* and *Aspergillus* are toxigenic fungi that produce the majority of fungal toxins, and their relative abundance significantly increases after sweating in RD (Figure 2K). After sweating, the relative abundance of *Penicillium* also exhibited an increase; however, no statistically significant difference was observed. These results indicate that the sweating process alters the fungal composition and increases the abundance of *Aspergillus and Fusarium* in RD.

Data are mean ± SEM (*n* = 3). *p < 0.05 and **p < 0.01 based on the independent-sample *t*-test.

### 3.3 Sweating process increases the contamination of mycotoxins in RD

**Figure 3:**
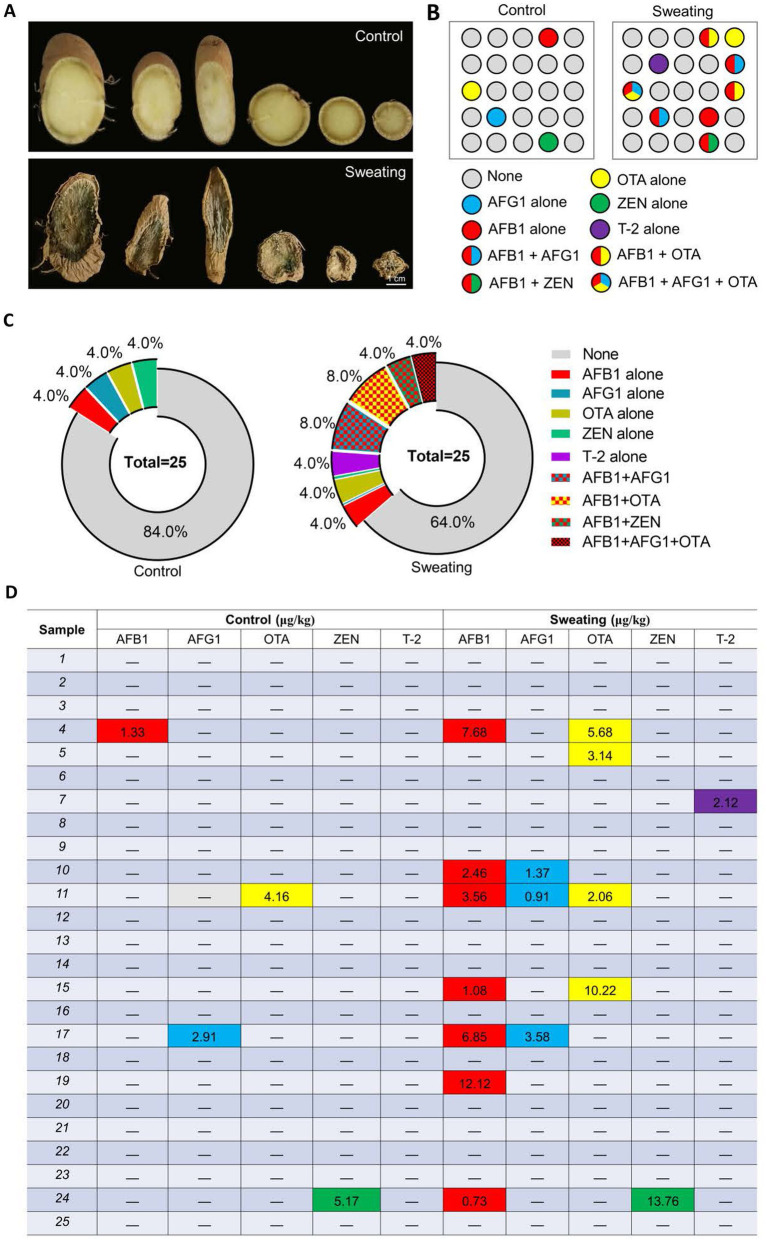
Detection of mycotoxins in RD before and after sweating process. **(A)** Changes in the appearance of RD before and after sweating process. **(B)** Changes in the number and type of mycotoxins-contaminated samples before and after sweating. **(C)** Percentages of samples contaminated with different mycotoxins, alone or in combination. **(D)** Levels of mycotoxin contamination of samples before and after sweating.

The sweating process caused significant changes in the appearance properties of the RD. The cross-section color of RD deepened with sweating, changing from faint yellow before sweating to atrovirens. The color change was the key feature of sweating treatment, and the content of terpenoids and phenolic acids is involved in the formation of green after sweating treatment (He et al., 2023). Additionally, fungal mycelium was visible on the surface in RD with sweating (Figure 3A). We further detected the mycotoxins in RD that were or were not subjected to sweating treatment using the UPLC-MS/MS method (Figure 3B). The results showed that the detection rate of AFs and OTs in the RD with sweating was significantly higher than that in the RD without sweating. Mycotoxins (AFB_1_, AFG_1_, OTA or ZEN) were detected in only 16% of RD samples without sweating, with contamination levels of AFB_1_, AFG_1_, OTA and ZEN were 1.33, 2.91, 4.16 and 5.17 μg/kg, respectively (Figures 3C,D). And there was no combined contamination of mycotoxins, suggesting that a small part of the RD may be infected by toxigenic fungus during cultivation. However, mycotoxins were detected in 36% of the samples with sweating. Among these samples contaminated with mycotoxins, 67% were contaminated with two or more mycotoxins. After sweating, the contamination levels of AFB_1_, AFG_1_, and OTA in the samples ranged from 0.73 to 12.12 μg/kg, 0.91 to 3.58 μg/kg, and 2.06 to 10.22 μg/kg respectively; while the contamination levels of ZEN and T-2 were 13.76 μg/kg and 2.12 μg/kg, respectively. Among these mycotoxin-contaminated samples, two samples had AFB_1_ levels exceeding 5 μg/kg, and one sample had both AFB_1_ and AFG_1_ levels exceeding 10 μg/kg. The ZEN contamination level in the sample was below 500 μg/kg, which meets the requirements of the Chinese pharmacopoeia (2020 edition) (Figures 3C,D). We also detected T-2 toxin in the RD with sweating, which was not detected in the RD without sweating (Figures 3B–D). These results suggested that the sweating process increases the contamination rate of mycotoxins and the risk of combined mycotoxin contamination in RD.

### 3.4 Isolation and identification of potential toxigenic fungi in RD

**Figure 4:**
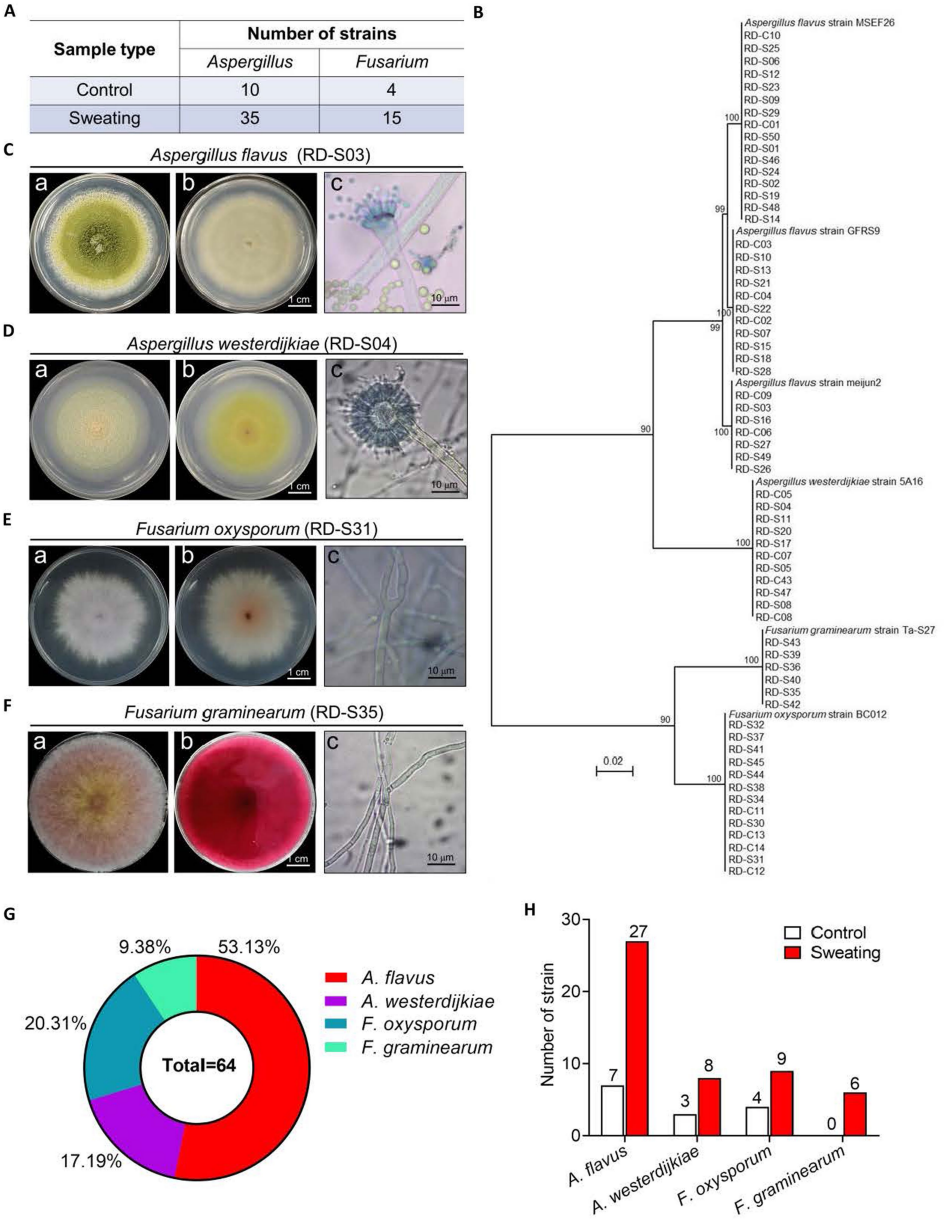
Morphological and molecular identification of potential toxigenic fungi from RD. **(A)** Number of *Aspergillus* spp. and *Fusarium* spp. isolated from RD that are or are not subjected to sweating treatment. **(B)** Phylogenetic tree based on gene sequences of specific fungi, inferred by using the Maximum Likelihood method, on 34 *A. flavus*, 11 *A. westerdijkiae*, 13F. *oxysporum* and 6F. *graminearum* isolated from RD, compared to reference sequences for corresponding species. **(C–F)** Morphological characteristics of *A. flavus, A. westerdijkiae, F. oxysporum* and *F. graminearum*. Inset **(A)** shows the front of the colony, inset **(B)** shows the back of the colony, and inset **(C)** shows the microscopic structure of the fungal hyphae and spores. **(G)** Percentages of *A. flavus, A. westerdijkiae, F. oxysporum* and *F. graminearum*. **(H)** The number of *A. flavus, A. westerdijkiae, F. oxysporum* and *F. graminearum* isolated from RD that are or are not subjected to sweating treatment.

We initially isolated 45 strains of *Aspergillus* and 19 strains of *Fusarium* from RD based on morphological and microscopic identification. Among them, there were 10 strains of *Aspergillus* and 4 strains of *Fusarium* in RD without sweating, while there were 35 strains of *Aspergillus* and 15 strains of *Fusarium* in RD with sweating (Figure 4A). The DNA of strains from RD was extracted and amplified using ITS1 and ITS4. Phylogenetic trees were constructed after comparing them with NCBI online BLAST. The results showed that 16 strains had a 100% similarity to *A. flavus* strain MSEF26, 11 strains had a 100% similarity to *A. flavus* strain GFRS9, 7 strains had a 100% similarity to *A. flavus* strain meijun2, 11 strains had a 100% similarity to *A. westerdijkiae* strain 5A16, and 6 strains had a 100% similarity to *F. graminearum* strain Ta-S27; additionally, there were also 13 strains had a 100% similarity to *F. oxysporum* strain BC012 (Figure 4B). Morphological and molecular identification confirmed the presence of potential toxigenic fungal strains in RD samples, including 34 strains of *A. flavus*, 11 strains of *A. westerdijkiae*, 13 strains of *F. oxysporum*, and 6 strains of *F. graminearum*, accounting for 53.13, 17.19, 20.13, and 9.38%, respectively (Figures 4C–G). The isolation frequency of *A. flavus*, *A. westerdijkiae* and *F. oxysporum* in RD with sweating was increased by 2.86-fold, 1.67-fold and 1.25-fold, respectively, compared to their isolation frequency from the RD without sweating (Figure 4H). In addition, we isolated 6 strains of *F. graminearum* in RD with sweating, which was not isolated in the RD without sweating (Figure 4H). This indicates that the sweating process will increase the number of endophytic toxigenic fungi in RD and produce new toxigenic fungi, increasing the risk of mycotoxin contamination.

### 3.5 Identification of key genes for mycotoxins biosynthesis in potential toxigenic fungi from RD

**Figure 5:**
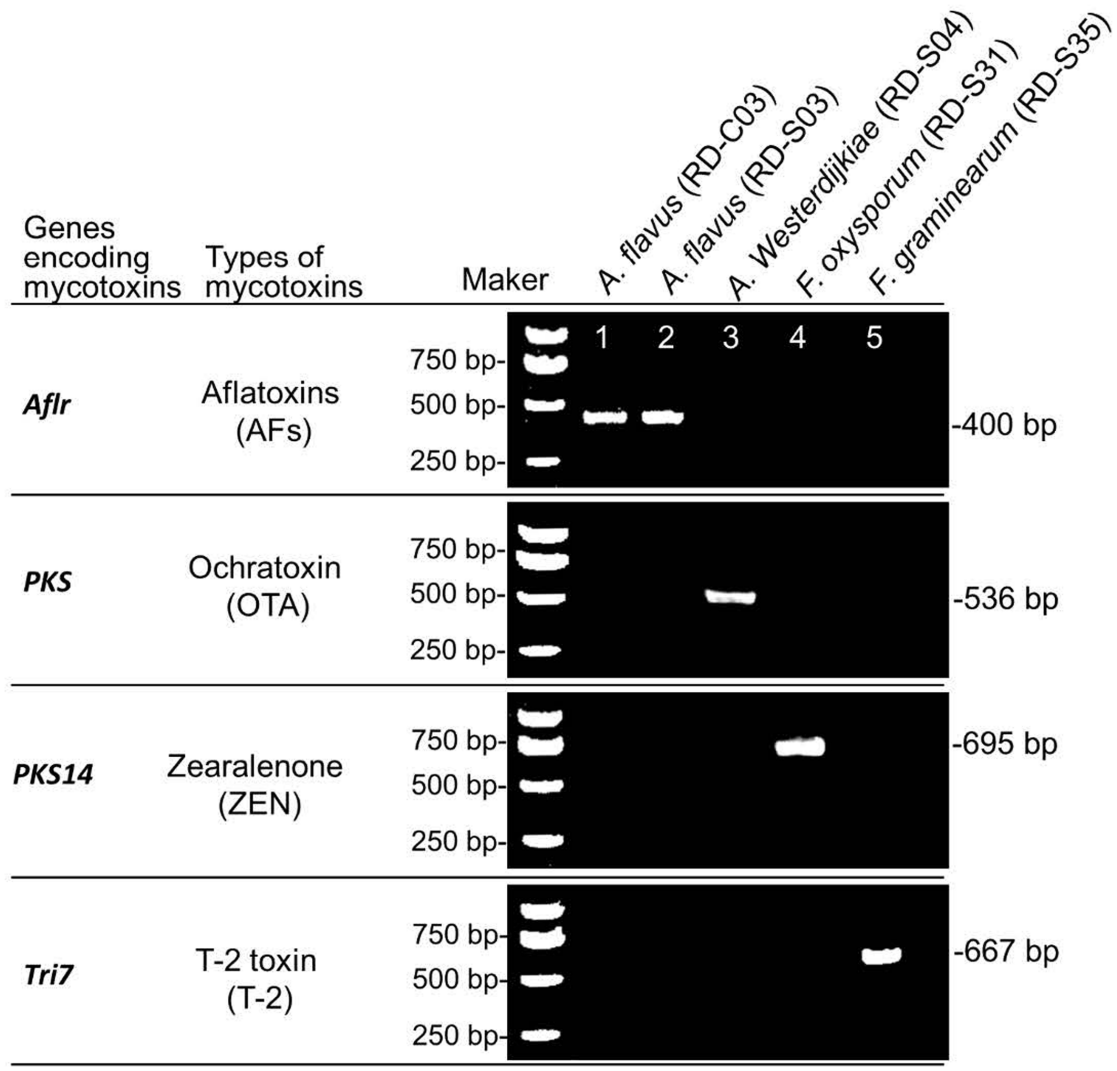
PCR identification of key genes for mycotoxins biosynthesis in potential toxigenic fungi from RD.

Using DNA from potential toxigenic fungi strains from RD as a template, PCR was used to detect the key genes for mycotoxin biosynthesis. The results showed that *A. flavus* (RD-C03 strain) and *A. flavus* (RD-S03 strain) carried *AflR*, which was the key gene for AFs biosynthesis. *A. westerdijkiae* (RD-S04 strain) carried *PKS*, which were key gene for OTs biosynthesis. *F. oxysporum* (RD-S31) carried *PKS14*, which was key gene for ZEN biosynthesis. *F. graminearum* (RD-S35 strain) carried *Tri7*, which was key gene for T-2 toxin biosynthesis (Figure 5).

PCR amplification and agarose gel detection of key genes for mycotoxins biosynthesis. M: DNA molecular weight standard; Lane 1–6: PCR products of key enzyme genes of toxigenic fungi strains were isolated.

### 3.6 Verification of the toxigenic capacity of each toxigenic fungus in different matrix

**Figure 6:**
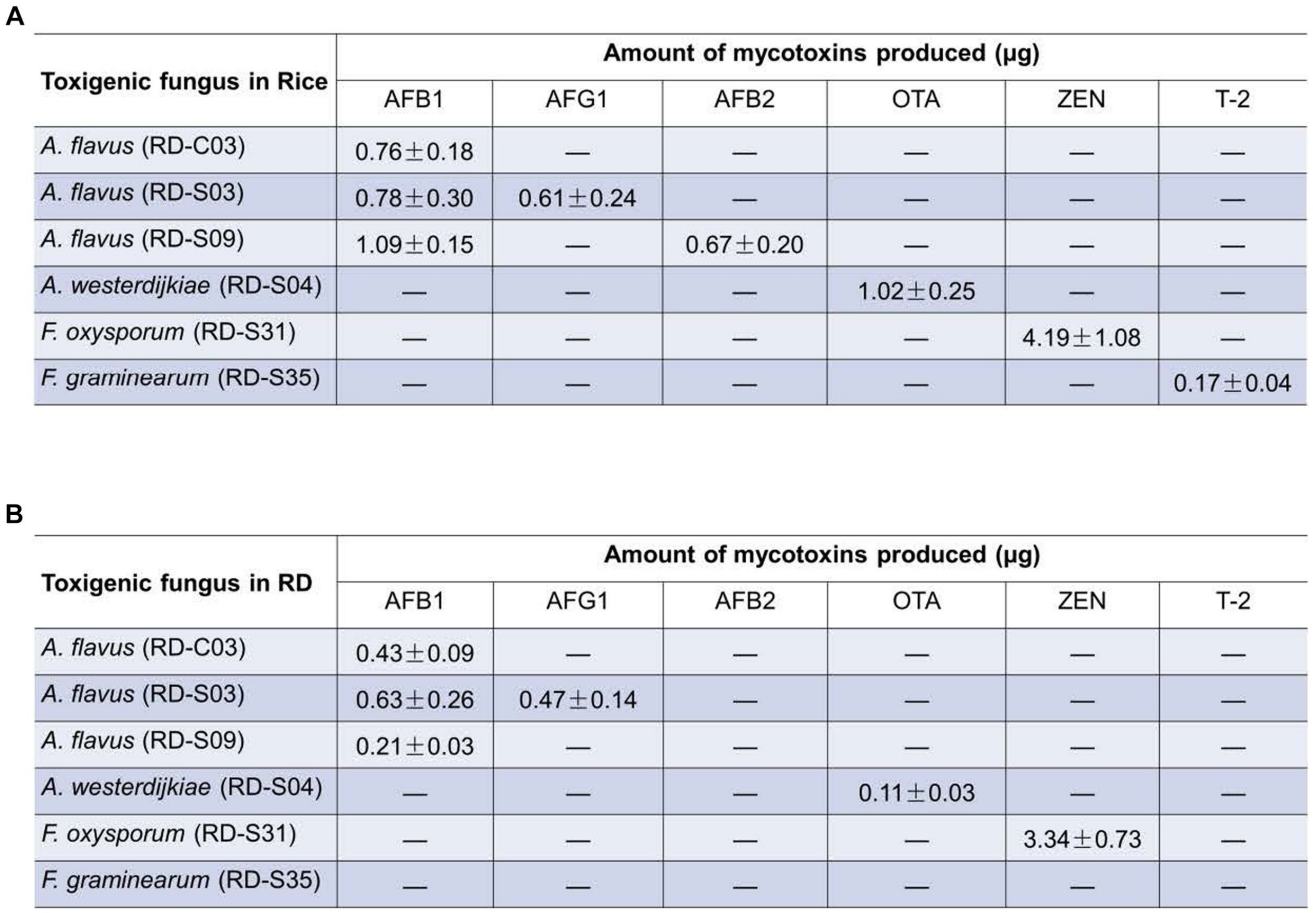
Amount of mycotoxins produced potential toxigenic fungi in **(A)** rice matrix or **(B)** RD matrix **(A,B)** the content of mycotoxins produced by toxigenic fungi in rice and RD matrix.

To determine whether these toxigenic fungi can produce their corresponding mycotoxins, we verified the toxigenic capacity of each fungus in different matrices using LC-MS/MS. The results showed that *A. flavus* (RD-S03 strain) could produce both AFB_1_ and AFG_1_ not only in rice matrix but also in the RD matrix (Supplementary Figures S1A,B). However, *A. flavus* (RD-C03 strain) could only produce AFB_1_, not AFG_1_, in either rice or RD matrix (Supplementary Figure S1A). *A. flavus* (RD-S09 strain) could simultaneously produce AFB_1_ and AFB_2_ in rice matrix, but it could only produce AFB_1_, not AFB_2_, in RD matrix (Supplementary Figures S1A–C). Both *A. westerdijkiae* (RD-S04 strain) and *F. oxysporum* (RD-S31 strain) can produce OTA and ZEN, respectively, not only in the rice matrix but also in the RD matrix (Figures 6A,B). *F. graminearum* (RD-S35 strain) can produce T-2 toxin in rice matrix, but not in RD matrix (Supplementary Figure S2C). The growth conditions of RD in the natural environment may be more complex, leading to other factors causing *F. graminearum* to produce T-2 toxin. The levels of mycotoxin production by *A. flavus*, *A. westerdijkiae*, *F. oxysporum*, and *F. graminearum* in the rice matrix and RD matrix were quantified (Supplementary Figures S2A,B).

## 4 Discussion

Sweating is one of the most important processing methods of Chinese medicinal herbs. However, the high temperature and humidity environment required for sweating Chinese medicinal herbs makes it very easy for fungi to breed, especially toxigenic fungi. The mycotoxins produced by these fungi will then contaminate the Chinese medicinal herbs. In this study, we demonstrated that the sweating process alters the fungal composition and increases the abundance of *Fusarium* and *Aspergillus* in RD, which in turn increases the contamination rate of mycotoxins and the risk of combined mycotoxin contamination in RD. Our data showed that the sweating process will increase the number of endophytic toxigenic fungi in RD and produce new toxigenic fungi, increasing the risk of mycotoxins contamination. To our knowledge, this study is the first to reveal an association between sweating processes and mycotoxins contamination of Chinese medicinal herbs.

Sweating is one of the most important processing methods for Chinese medicinal herbs, which promotes the enzyme system and initiates the biological and chemical transformation of primary/secondary metabolites (Cao et al., 2020; Sun et al., 2023). Then the changes in biochemical properties, metabolite levels, and medicinal properties of medicinal herbs are altered (Wang et al., 2016). Our study demonstrated that sweating can alter the color of the fracture surface of RD from pale yellow to dark green, which is correlated with the levels of terpenoids and phenolic acids (He et al., 2023). However, the high temperature and humidity environment required for the sweating of Chinese medicinal herbs makes it very easy for toxigenic fungi to breed (Zhang et al., 2018; Locatelli et al., 2022). Indeed, our results showed that the sweating process altered the fungal composition and increased the abundance of *Aspergillus* and *Fusarium* in RD, which is consistent with a previous report. The investigation revealed significant differences in the contaminated fungal communities of Cassia seeds with different processing specifications (Guo et al., 2020). The relative abundance of *Aspergillus* in roasted Cassiae Semen was higher than that in raw Cassiae Semen, and *Aspergillus*, *Fusarium* and *Penicillium* were mainly distributed in burnt malt (Long et al., 2022). *Aspergillus* are the toxigenic fungi responsible for most AFs and OTs (Cabrera-Meraz et al., 2021; Kumar et al., 2023), and *Fusarium* are the toxigenic fungi responsible for most fusarium mycotoxins, such as deoxynivalenol, ZEN and so on (Narvaez et al., 2020; Ji et al., 2023).

The relative abundance of toxigenic fungi *Fusarium* and *Aspergillus* increased after the sweating process, possibly because the humid and hot environment created by sweating is more conducive to the growth of these fungi. Temperature and water and their interactions have been shown to be key factors that regulate fungal growth and secondary metabolite production (Schmidt-Heydt et al., 2009; Marin et al., 2010). In studies on aflatoxins, these two conditions not only promote the growth of aflatoxin-producing fungi (mainly aflatoxins) but also have a significant impact on the activation of gene clusters responsible for aflatoxin production (Abdel-Hadi et al., 2012; Tejero et al., 2021). It was reported that *A. flavus* could not grow under conditions of temperatures below 10°C in the culture medium of Chaezella yeast AGAR (CYA) and corn extract (CEM), indicating that the lowest temperature for *A. flavus* growth was between 8–10°C (Astoreca et al., 2012). The lowest temperature for *A. flavus* growth on the PDA culture medium was 12°C (Calvo et al., 2002), and on rice, it was 13.2°C (Mousa et al., 2011). Higher water activity facilitates better fungal growth and toxin synthesis (Bhatnagar et al., 2006). AFB_1_ production can be detected when the water activity is 0.94–0.99, and aflatoxin production is inhibited when the water activity is lower than 0.94 (Lahouar et al., 2016). In our study, we found that the sweating process caused a significant decrease in the Ace, Chao, and Shannon indices of fungi in RD, indicating a reduction in the richness and diversity of fungal species. This reduction in microbial diversity and richness in RD with sweating may be associated with the inhibitory effect of toxigenic fungi on non-toxigenic fungi (Martinez-Culebras et al., 2021; Khalifa et al., 2022). Numerous studies have demonstrated that mycotoxins, such as AFs, OTs, or ZEN, can hinder the growth of other fungi by disrupting membrane structure and metabolism (Zheng et al., 2019; Rodrigues et al., 2021). In the present study, we detected more mycotoxins in the RD samples with sweating. Additionally, the detection rate of AFB_1_, AFG_1_, OTA or ZEN in the RD with sweating was significantly higher than that in the RD without sweating. It is worth mentioning that toxigenic fungi were also isolated, and mycotoxins were detected without sweating RD. This result suggests that the RD carried toxigenic fungi before sweating, but the population of toxigenic fungi expanded after sweating. Root herbs are more susceptible to contamination by fungi and mycotoxins due to direct contact between their roots and the soil (Su et al., 2018). After the introduction of toxigenic fungi from soil to herbal medicines, these fungi may proliferate and produce mycotoxins during processing and storage (Su et al., 2018). Aspergillus fungi exist widely in nature, can adapt to the low water environment, and are the dominant fungus in the fungal pollution of medicinal materials (Rizzo et al., 2004; Wei et al., 2023). The roots of RD are rich in lignin and fiber. It is speculated that a variety of extracellular enzymes (pectinase and cellulase) secreted by *Aspergillus* fungi can effectively decompose lignin and fiber and provide a carbon source for growth. The types and contents of nitrogen sources and carbon sources in the substrate have a great influence on the growth and toxicity of fungi (Chang et al., 1995; Feng and Leonard, 1998; Ehrlich and Cotty, 2002). *Fusarium* fungi are also poisonous fungi in RD. The results showed that *Fusarium* fungi were suitable for high water and abundant carbon source environments, and had weak growth and toxic production ability in herbs with low water and poor carbon source (Rodrigues et al., 2021).

Simultaneous detection of multiple mycotoxins in our study also showed that about 67% of the mycotoxin-contaminated RD with sweating were contaminated by 2–3 mycotoxins. However, this combined contamination of mycotoxins was not found in the RD without sweating. The possible reason for this phenomenon is that toxigenic fungi can transmit to each other among medicinal herbs during sweating, leading to the expansion of contamination and resulting in multiple mycotoxin combinations (Wang et al., 2020). According to the 2020 edition of the Chinese Pharmacopoeia, AFB_1_ limit is set at 5 μg/kg for 24 Chinese herbs, while the total AF should not exceed 10.00 μg/kg and ZEN content should be below 500 μg/kg. Three of the samples that were contaminated with mycotoxins after sweating had aflatoxin levels that exceeded the limit. These results suggested that sweating process increased the contamination rate of mycotoxins and the risk of combined contamination of mycotoxins in RD. We also detected T-2 toxin in the RD with sweating, which was not detected in the RD without sweating. Studies have shown that T-2 toxins exhibit basic toxicological characteristics such as immunotoxicity, cytotoxicity, DNA damage, blood toxicity, reproductive toxicity, and hepatorenal toxicity (Li et al., 2011). These results suggested that the process of sweating can also introduce exogenous toxigenic fungi into the herbs, implying that the sweating environment may also be responsible for toxigenic fungal contamination. It has been reported that in the coexistence mode of multiple mycotoxins, their toxic effects are far more complex than simply adding up the toxicity of a single toxin. Additionally, there may be antagonistic, additive, and synergistic effects among mycotoxins. For instance, the combination of AFB_1_ and OTA can enhance its toxicity (McKean et al., 2006; Xu et al., 2022). The combination of AFB_1_ and T-2 increases their toxicity (Hou et al., 2018; Xiao et al., 2023). In Vero kidney cells, AFB_1_ and OTA not only exhibit cumulative cytotoxic effects but also demonstrate synergistic effects that enhance genotoxicity by increasing DNA fragmentation (Golli-Bennour et al., 2010). The effects of AFB_1_, DON, T-2, and ZEN on cells are dose-dependent and time-dependent (Calvert et al., 2005). Combined exposure to AFB_1_ and ZEN during pregnancy and lactation in rats caused severe toxicity to the mammary gland and liver. High doses of ZEN exacerbated the toxicity of AFB_1_, while low doses of ZEN alleviated the damage caused by AFB_1_ (Wu et al., 2022). The above research demonstrates that the toxicity of multiple mycotoxins in combination is stronger than that of a single toxin. In this study, RD medicinal materials were contaminated by various toxins, which poses a certain risk to the safety of RD drugs. Therefore, it holds significant importance for controlling mycotoxin pollution during the processing of RD.

Currently, various types of toxigenic fungi have been isolated and identified from numerous Chinese medicinal materials. Researchers discovered that all strains of *Penicillium polonicum*, which were isolated from OTA-contaminated licorice root, produced OTA (Chen et al., 2011). Some researchers isolated and identified toxic fungi mainly from wolfberry, Angelica, and licorice (Zheng et al., 2017). This study also isolated and identified toxigenic fungi from RD. We found that *A. flavus* was the main source of aflatoxins in RD, while *A. westerdijkiae* was responsible for producing ochratoxins in RD. The RD-S03 strain of *A. flavus* was capable of producing both AFB_1_ and AFG_1_ in rice and the RD matrix. This may be because AFB_1_ and AFG_1_ have the same dihydrodifuran structure, and their synthetic pathways are similar (Yabe et al., 1988). The RD-S09 strain of *A. flavus* can also produce two toxins, AFB_1_ and AFB_2_, in rice medium. However, AFB_2_ is not detected in the RD matrix, which could be due to the more complex growth environment of toxigenic fungi in natural settings. The RD matrix may be lacking a certain gene enzyme that promotes the synthesis of AFB_2_. Toxigenic fungi can grow on various substrates, but different substrates have a greater impact on toxin production. Some substrates are conducive to mycotoxin production, while in others, toxic fungi can grow and reproduce in large numbers but produce very low amounts of toxins or no toxins at all (Wellford et al., 1978; Llewellyn et al., 1981). Sherertz et al. (1976) investigated the production of toxicity by different aflatoxin-producing fungus in raw and cooked soybeans, revealing that the optimal substrate for toxin production varied among different toxigenic fungus producing the same toxin. We found that ZEN in RD was mainly derived from *F. oxysporum*, while T-2 toxin was produced by *F. graminearun* in rice matrix but not in RD matrix. This may be due to the more complex growth conditions of RD in its natural environment. There are other factors that cause *F. graminearun* to produce T-2 toxin, and medicinal materials are not the optimal substrate for the growth and production of toxigenic fungi. Compared to grain substrate, the growth of toxigenic fungi may be hindered to some extent due to the lack of readily available carbon and nitrogen sources and the fungicidal function of certain components in medicinal materials (Llewellyn et al., 1981), which could explain why no mycotoxins were detected in RD matrix.

## 5 Conclusion

Our results suggested that the sweating process increased the expansion of toxigenic fungi from *Fusarium* and *Aspergillus* in RD, which in turn increases the contamination rate of AFs, OTA, ZEN, and T-2 toxin as well as the risk of combined mycotoxins contamination in RD. This study reveals an association between the sweating process and the contamination of mycotoxins in RD, providing a basis for preventing the contamination of toxigenic fungi during the sweating process of Chinese medicinal herbs.

## Data availability statement

The datasets presented in this study can be found in online repositories. The names of the repository/repositories and accession number(s) can be found below: https://www.ncbi.nlm.nih.gov/bioproject/PRJNA1085876/.

## Author contributions

YG: Methodology, Writing – review & editing, Investigation, Validation, Writing – original draft. LW: Investigation, Methodology, Validation, Writing – review & editing, Software. DS: Investigation, Methodology, Validation, Writing – review & editing. QY: Writing – review & editing, Visualization. CX: Writing – review & editing, Data curation. MH: Writing – review & editing, Investigation. CK: Writing – review & editing, Software. LG: Writing – review & editing, Conceptualization, Supervision. TZ: Writing – review & editing, Project administration, Resources. JZ: Project administration, Writing – review & editing, Conceptualization, Methodology, Supervision.

## Footnotes

^1^ https://blast.ncbi.nlm.nih.gov/Blast.cgi
