# Supplementary material for: The sweating process promotes toxigenic fungi expansion and increases the risk of combined contamination of mycotoxins in Radix Dipsaci
Source: Front Microbiol. 2024 Jun 6;15:1394774. doi: 10.3389/fmicb.2024.1394774 (PMC11187008; doi:10.3389/fmicb.2024.1394774)
Supplement: Supplementary file 1 [file Data_Sheet_1.PDF]

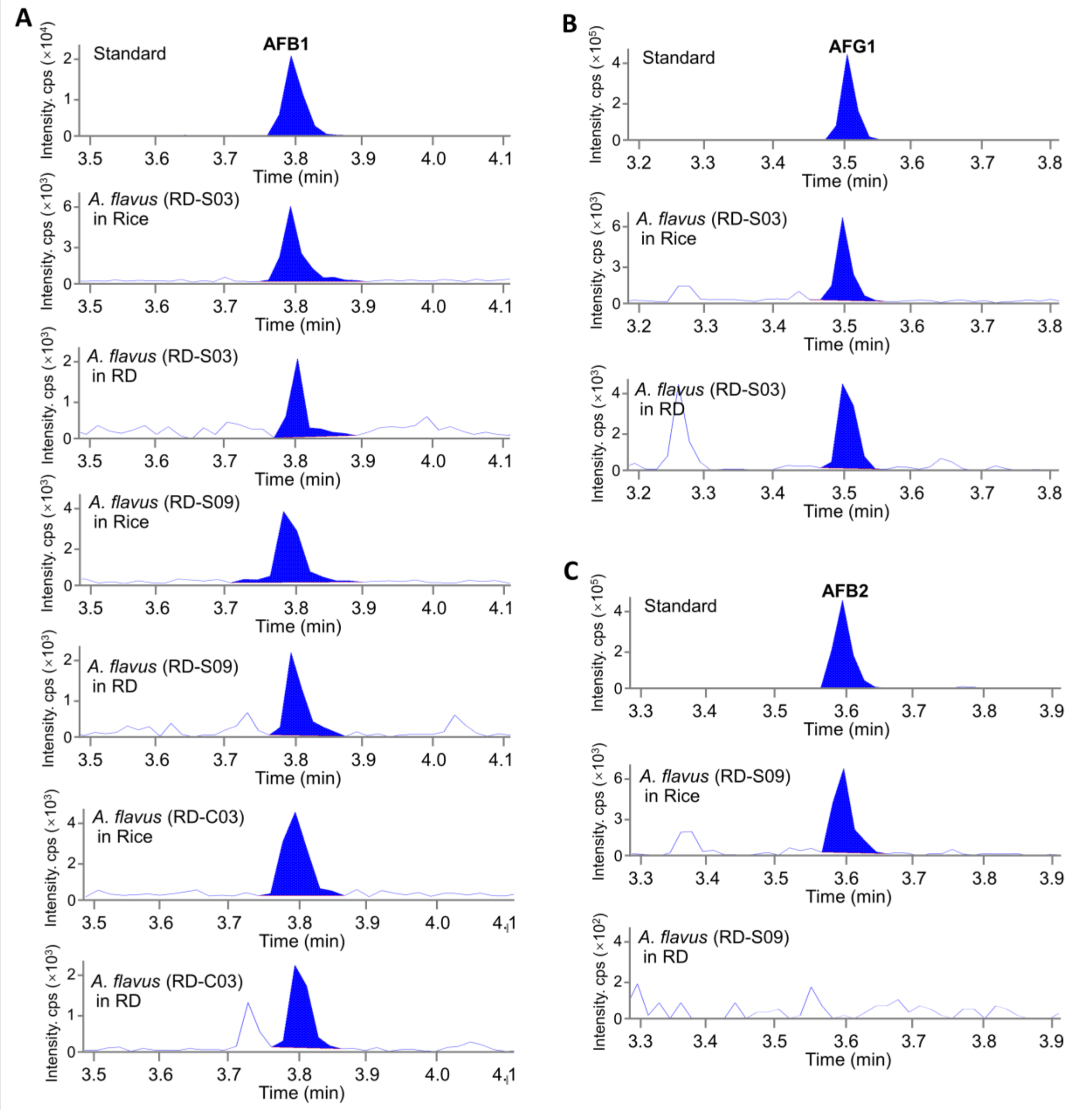

Supplementary figure 1. Verification of aflatoxins production in potential toxigenic fungi from RD contaminated with mycotoxins

(A-C) The chromatogram of mycotoxins produced by toxigenic fungi in rice and RD matrix.

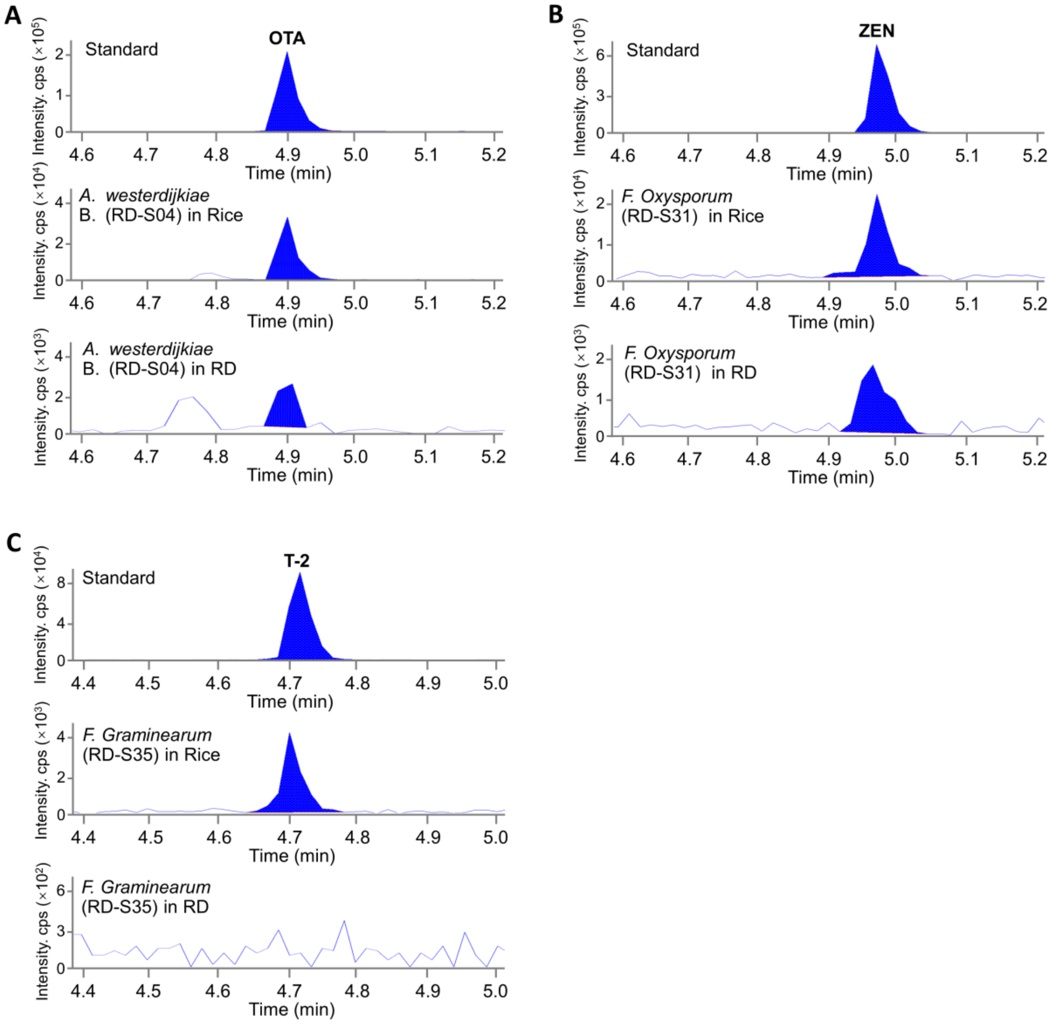

Supplementary figure 2. Verification of ochratoxin (OTA), zearalenone (ZEN) and T-2 toxin (T-2) production in potential toxigenic fungi from RD contaminated with mycotoxins

(A-C) The chromatogram of mycotoxins produced by toxigenic fungi in rice and RD matrix.
